# DBS Programming: An Evolving Approach for Patients with Parkinson's Disease

**DOI:** 10.1155/2017/8492619

**Published:** 2017-09-24

**Authors:** Aparna Wagle Shukla, Pam Zeilman, Hubert Fernandez, Jawad A. Bajwa, Raja Mehanna

**Affiliations:** ^1^Center for Movement Disorders and Neurorestoration, Department of Neurology, University of Florida, Gainesville, FL, USA; ^2^Center for Neurological Restoration, Cleveland Clinic, Cleveland, OH, USA; ^3^Parkinson's Disease, Movement Disorders and Neurorestoration Program, National Neuroscience Institute, King Fahad Medical City, Riyadh, Saudi Arabia; ^4^University of Texas Health Science Center, McGovern Medical School, Houston, TX, USA

## Abstract

Deep brain stimulation (DBS) surgery is a well-established therapy for control of motor symptoms in Parkinson's disease. Despite an appropriate targeting and an accurate placement of DBS lead, a thorough and efficient programming is critical for a successful clinical outcome. DBS programming is a time consuming and laborious manual process. The current approach involves use of general guidelines involving determination of the lead type, electrode configuration, impedance check, and battery check. However there are no validated and well-established programming protocols. In this review, we will discuss the current practice and the recent advances in DBS programming including the use of interleaving, fractionated current, directional steering of current, and the use of novel DBS pulses. These technological improvements are focused on achieving a more efficient control of clinical symptoms with the least possible side effects. Other promising advances include the introduction of computer guided programming which will likely impact the efficiency of programming for the clinicians and the possibility of remote Internet based programming which will improve access to DBS care for the patients.

## 1. Introduction

Deep brain stimulation (DBS) therapy was approved by the US Federal Drug Administration (FDA) in the year 2002 for treatment of motor symptoms in Parkinson's disease [1]. The efficacy of DBS has been well established through randomized controlled studies involving several hundreds of Parkinson's disease patients [2]. DBS is effective for control of tremors that are refractory to dopaminergic medications, motor fluctuations, and levodopa induced dyskinesia that are bothersome to patients. The success of DBS is dependent on many factors including selection of appropriate patients, accurate placement of DBS lead, and a thorough programming process to identify the optimal stimulation parameters [3]. Selection of appropriate patients is based on many factors including the age of the patient, disease stage, disease duration, comorbidities, and responsiveness to levodopa medication. These factors are discussed by an interdisciplinary team consisting of neurologist, neurosurgeon, psychiatrist, neuropsychologist, rehab specialist, and sometimes a social worker. Once the DBS lead is placed in an appropriate target using standard surgical technique, DBS programming is initiated which in most cases is a time and labor intensive manual process involving multiple patient visits [4]. DBS programming is generally performed by movement disorder neurologists (including fellows in training), neurosurgeons, nurses, nurse practitioners, or physician assistants who have acquired training and experience for this procedure. Although there are general guidelines available for programming, there are no clear, validated, and established programming protocols. An inefficient programming can result in suboptimal clinical outcomes and lead to side effects which becomes a source of frustration for Parkinson's disease patients and caregivers as well as healthcare providers [3]. These patients are then referred to as “DBS failures” and referrals are placed to advanced DBS centers for consideration of a lead revision surgery. In a retrospective analysis of 41 patients who presented to two academic DBS centers for management of “DBS failures,” over a period of two years, 15 patients (37%) were identified as inadequately programmed and they improved significantly after reprogramming. There were 6 additional patients (15%) who benefitted partially from expert reprogramming, and 21 patients (51%) failed to improve despite a detailed reprogramming. There were also seven (17%) patients who did not demonstrate clinical improvement due to poor access to programming [5]. Thus lead revision is potentially avoidable when a careful and systematic algorithm based programming is employed.

## 2. Current Approach to DBS Programming

### 2.1. Initiation of Programming

In Parkinson's disease, a successful DBS programming is usually accomplished over a period of three to six months. Programming is usually not initiated immediately after the placement of a lead; instead a time frame of 2–4 weeks is allowed for the microlesion effects to fade away. These microlesion effects are believed to arise from the trauma of the DBS lead implantation rather than from the stimulation of the targeted brain structure. As a result, there is temporary improvement in clinical symptoms. Thus, for an accurate assessment of stimulation benefits, it is recommended that DBS programming gets initiated only when the initial benefits fade away [6]. In a large randomized controlled DBS study, the mean medication “on” time in patients randomized to receive delayed stimulation therapy was observed to improve at three months after surgery attributed to the microlesion effects. Nearly 40% of this group responded with an improvement of more than 2 hours of “on” time compared to the case before surgery [7]. There are some DBS centers that advocate initiation of programming at an earlier stage while the patients are still hospitalized as this method is more patient convenient and avoids an extra programming visit [8]. In addition to the microlesion effect confounding the initial results, impedance fluctuations in the tissue surrounding the DBS lead can also contribute to inaccurate assessment. Impedances are observed to be increased immediately after placement of a lead, as a consequence of edema, and they tend to decrease and stabilize over the first few weeks [9]. In these situations, DBS therapy delivered through constant-voltage stimulation is avoidable as the current delivered depends on the impedance. Instead, a constant-current stimulation that allows the current to adapt to changes in the impedance is recommended.

### 2.2. Lead Type, Impedance Check, Programming Thresholds, and Battery Check

In order to utilize effective stimulation parameters at the bedside, it is important for the DBS programmer to be aware of the lead type which refers to the size of the contacts and the distance between them. With the Medtronic system, the commonly used lead models are the 3387 and the 3389. The 3387 model is a 40 cm long and 1.27 mm wide cylindrical lead with 4 cylindrical electrodes that are 1.5 mm in length each and placed 1.5 mm apart. The 3389 model carries the same specifications except for electrode spacing of 0.5 mm. The Boston Scientific DBS lead has 8 cylindrical contacts that are 1.3 mm in diameter and 1.5 mm in length, placed 2 mm apart and covering a span of 15.5 mm. The Boston Scientific DBS system also offers a directional lead in which the middle two levels are split into three segments spanning approximately 120 degrees and the highest and the lowest level contain ring shaped electrodes. The Boston Scientific system is currently not FDA approved; however trials are underway. The St Jude Infinity DBS system (now called Abbott's Infinity DBS system) that has segmented electrodes and a wireless mobile platform for programming recently received FDA approval.

It is also necessary to confirm the location of the DBS lead prior to initiation of programming. At our center, we routinely obtain a postoperative CT brain that is coregistered with the preoperative MRI scan. Another important step is to gather intraoperative records for review of stimulation parameters used for testing immediately after the implantation. Once these steps are completed, the programming healthcare professional records the impedance at each of the contacts to establish a baseline for future reference. Compared to intraoperative parameters (influenced by edema), the impedance recorded is often different. If an impedance recording suggests a short circuit or an open circuit then the impedance is rechecked at higher voltages to ensure accuracy of the reading. The older Soletra® and Kinetra® Medtronic models require the provider to manually select the higher voltage for the repeat impedance check, whereas the Activa® SC/RC/PC will automatically check at 1.5 V and 3.0 V if open circuit is noted at 0.7 V stimulation. If there is a short circuit (which is extremely low impedance < 250 ohms) then the provider is not required to check at higher voltages. When a short circuit is identified, it is recommended to avoid the involved contacts as these are not dependable. There is generally faster battery depletion or there is sometimes a sudden loss of benefit. A common reason identified for short circuit has been anchoring of DBS lead with a miniplate [10]. High impedance, for example, 2000 ohms for the Soletra and 4000 ohms for the Kinetra, should be in general seen concurrently with unipolar and bipolar review. If the impedances are high in the bipolar contacts but normal in the unipolar contacts then there may not be an open circuit. Decisions regarding open circuit findings need to be evaluated on a case-by-case basis. The high impedance (open circuit) will be generated in the Activa SC/PC/RC when >10000 ohms in unipolar and bipolar configuration is noted [11]. The St Jude DBS system will show a message of “high” (read as 31 with older version and with newer one as >3000) when there is an open circuit. Lead fractures are common reasons for open circuits with an overall incidence of 5.1%, clinically presenting as electrical shocks reported by patients or lack of a therapeutic benefit. In the context of Parkinson's disease, it is also important to consider head jerking from cervical dystonia or a twiddler's syndrome, in which the patients who have developed dopaminergic medication induced impulse control disorder subconsciously spin the neurostimulator in the chest wall which results in lead fractures [12]. In a series of 226 DBS patients, three patients identified to have a twiddler's syndrome presented with reemergence of Parkinson's disease symptoms and pain along the path of the hardware. In these patients, twisting/fracture of DBS extension was identified radiographically and was treated surgically by securing the neurostimulator in the chest wall [13, 14].

Once the electrical intactness of the system is established, thresholds of stimulation parameters that elicit benefits and induce side effects are determined. Initially, each electrode contact on the lead is tested in a monopolar configuration with the electrode as negative (cathode) and the neurostimulator case as positive (anode), a process referred to as monopolar review. The main stimulation parameters include the voltage, the frequency, and the pulse width. Amplitude controls the intensity of the stimulation, pulse width refers to the duration of each electrical pulse delivered, and frequency is the rate of stimulation employed in programming. The Medtronic system for the Soletra and Kinetra is only available in amplitudes of voltage (V). The Medtronic system for the Activa SC/RC/PC is available in either amplitudes of V or milliamps (mA). The St Jude and Boston Scientific systems are available only in amplitudes of mA. With a fixed frequency and pulse width, each of the electrode contacts is separately examined with amplitude delivered at increasing increments of 0.5 V or mA until there is elicitation of adverse effect (objective or subjective) that stays persistent with continued stimulation. This establishes a stimulation threshold for the adverse effects. Then the efficacy of stimulation at this contact is examined using an amplitude reduction by 0.1–1.0 V or mA below the stimulation threshold for side effects [15]. As the amplitude is reduced, the lowest threshold for inducing the best clinical benefits is determined. On the other hand, some centers first identify the threshold for clinical benefit and then increase the amplitude to identify the threshold for side effects. The electrode contact with the widest therapeutic window (wider difference between the threshold for inducing side effects and the threshold for clinical benefits) is selected for chronic stimulation. Both clinical effects and side effects depend on the direction of spread of current stimulating the anatomical structures as described in detail in the Table 1. If there is inadequate control of motor symptoms with single monopolar configuration, the next choice is to employ double monopolar stimulation with the two stimulation contacts as negative and the neurostimulator case as positive. There is no fixed time interval on taking this decision but most programmers wait few weeks or couple of programming sessions before switching to a bipolar configuration. Alternate method is to stay in monopolar stimulation but adjust the frequency or the pulse width. Bipolar configuration (most effective contact is negative and the adjacent contact is positive) is sought if side effects with monopolar configuration are induced at low amplitudes. With bipolar configuration, higher stimulation intensities are sometimes required to achieve the same clinical benefit.

In theory, DBS programming for a Medtronic device involves thousands of possible parameter combinations considering the range of programmable amplitudes (>90 possible), pulse widths (>10 possible), frequencies (>25 possible), interleaving settings, and configuration of anodes and cathodes. However since the recommended limit for charge density is 30 mC/cm^2^ which is calculated by dividing the product of the voltage and the pulse width by the product of the impedance and the geometric surface area of the DBS electrode (0.06 cm^2^) it limits the number of possible combinations [16]. There is a wide variation in the final stimulation parameters selected for DBS programming which is driven by multiple factors such as patient characteristics, the specific Parkinson's disease phenotype, and the lead position. In Parkinson's disease, the stimulation parameters used with a Medtronic system consist of a range of pulse widths (60 to 450 *μ*s), frequencies (60 to 160 Hz), and voltages or currents (1 V to highest tolerated value). In most clinical DBS studies for Parkinson's disease, voltage in the range 2.4 to 4.4 V, frequency in the range 143 to 173 Hz, and pulse width in the range 67 to 138 *μ*s have been found to effectively control the motor symptoms [17]. For efficient management of motor symptoms few published algorithms are available. In one study from Grenoble, France, with several combinations of stimulation settings that were systematically evaluated in patients with Parkinson's disease, the most important factors for alleviation of motor symptoms were identified as the voltage followed by the frequency [18]. In a recent study, an algorithm was proposed to specifically address the speech issue, gait impairment, and stimulation induced dyskinesia. The authors suggested lowering of stimulation frequency once other considerations including reduction of voltage, stimulation with bipolar configuration, and interleaving pattern had been tried with no clinical improvements. Caution should be exercised while using low frequency DBS as there is a possibility of worsening of appendicular rigidity, bradykinesia, and tremor [3].

### 2.3. Programming Visits

The initial programming visit can be often long lasting nearly 60–90 minutes. During this visit, it is important to provide patient education on several matters that are pertinent for a successful DBS programming. These include the knowledge on potential stimulation induced side effects, the use of the patient programmer (how to turn on and turn off the stimulator or go between patient group settings or programs if provided or adjusting parameters provided to the patient), and the safety precautions that need to be followed such as avoidance of strong magnetic fields and the use of diathermy during surgical procedures. Thus, family and friends are encouraged to accompany the patients during this initial programming visit. The programming is usually performed in the morning in an off-dopaminergic medication state. The rationale for holding medications is that dopaminergic medications can potentially obscure the stimulation induced benefits. Patients are instructed to hold the medications overnight or to miss at least a couple of doses so that they present to clinic in the “off-” medication state. If off-medication symptoms are intolerably severe or there is a lack of family support for outpatient management, inpatient programming is recommended. Alternately, patients are allowed to present in an on-medication state and they are examined once the medications effects show signs of wearing off (suboptimal on-medication) [17]. Standardized motor tasks of the Unified Parkinson's Disease Rating Scale are used for clinical assessment. Amongst all the cardinal motor symptoms of Parkinson's disease, tremors and rigidity are found to respond very quickly, usually within seconds to minutes of stimulation, whereas there is a variable time delay for improvement in bradykinesia. The clinical response to DBS depends on several factors such as disease characteristics, DBS lead position, stimulation parameters, and individual patient profile. Since patient participation is critical, factors such as patient fatigue, patient comfort, patient anxiety, and training contribute significantly to the outcome.

Once the off-medication state programming is completed, patients are given their usual dopaminergic dose to further determine stimulation parameters for control of levodopa induced dyskinesia. It is noteworthy that levodopa induced dyskinesia does not necessarily emerge immediately after the first dose of medication, sometimes requiring the cumulative effects of two or three doses to develop, and is most often seen in the afternoon. The best electrode configuration is the one that adequately improves off-medication parkinsonism and reasonably suppresses on-medication dyskinesia. A challenge that arises in relation to subthalamic nucleus DBS is stimulation induced dyskinesia when the DBS lead is well-positioned in the motor territory. In these circumstances, a gradual reduction of stimulation voltage is recommended to achieve balance between control of parkinsonism and control of dyskinesia. On the other hand, stimulation of the dorsal globus pallidum may show differential effects on control of dyskinesia based on the specific anatomical region stimulated. There are reports that stimulation of dorsal globus pallidus internus induces dyskinesia which may be confused with medication related dyskinesia. When stimulation contact is shifted ventrally, dyskinesia becomes suppressed and bradykinesia tends to worsen [19].

DBS programming requires multiple patient visits. During the initial six months after surgery, patients are followed every month. Once the optimal programing settings are determined, patients are then followed on an annual basis for clinical performance, troubleshooting, and battery checks. An earlier follow-up is scheduled if the disease status worsens at a faster pace.

### 2.4. Battery and Programming

During the follow-up of DBS patients, estimation of battery life is critical. Battery drain is dependent on many factors including manufacturing tolerances, battery usage, battery chemistry, and variations in tissue impedance. The electrode surface area (small surface areas result in larger impedances) and the number of contacts used for stimulation affect the tissue impedance [20, 21]. With the Medtronic Soletra system, the battery life starts at a voltage of 3.69–3.74 V with an end of life (EOL) reached when the battery drains to about 2.5 V. In general with Soletra battery the voltage stays the same over a period of time; however as the battery nears the end of longevity, a slow drop in voltage may occur followed eventually by a more rapid depletion. Some patients notice worsening of symptoms when the battery is depleting and thus waiting to reach 2.5 V is not necessary to plan replacement of the DBS battery. With the Medtronic Kinetra system, the starting battery voltage is 3.2 V and the EOL is reached around 1.97 V. The Kinetra battery voltage reading slowly decreases over time; sometimes the Kinetra battery will stop showing a decline in the battery voltage for several visits; however if the patient complains of return in symptoms then it is important to make plans to replace the DBS battery. The current consumption with Kinetra is linear, unlike Soletra where the voltage doubler or tripler circuit is activated once the voltage parameter delivered for clinical stimulation increases to 3.6 V leading to a faster drain of battery [22]. In addition to the battery status indicator available in each device, battery life can be estimated through helplines/website made available locally or through Medtronic Inc [20]. Newer generation DBS systems offer rechargeable neurostimulators such as the Activa RC through the Medtronic (expected lifespan of about 9 years) or Vercise® system through the Boston Scientific (expected lifespan of about 25 years). The Abbott Infinity® system has a battery life of 3–5 years (Saint Paul, MN, USA) [23]. Medtronic Activa RC, Boston Scientific Vercise (not approved by FDA yet), and Abbott Brio all offer rechargeable DBS batteries. The Abbott Infinity 5 and Infinity 7 batteries show a status of either “battery okay,” “battery low,” or “battery depleted.” Further details on battery life and impedance details are provided in Table 2. Future advances in DBS technology such as closed loop DBS will increase battery life and advances in DBS programming like remote and Internet based programming will increase patient comfort and convenience [24].

## 3. Recent Advances in DBS Programming

The electrical field delivered through the DBS contact in monopolar configuration is spherical with intensity of field decreasing in proportion to distance from the electrode. Large diameter myelinated axons have the lowest threshold for activation compared to dendrites and soma and also respond to shorter pulse widths. With bipolar configuration, the intensity of field decreases to one-quarter when the distance from electrode doubles. The intensity of electrical field increases as the distance between cathode and anode increases with wider bipolar configuration giving higher intensity field compared to narrow configuration. The conventional DBS however has limited capabilities with regard to modulating the shape of electrical field and tailoring the intensity of stimulation to maximally stimulate the neuronal pathways of interest and minimize the unintended spread to anatomical structures leading to side effects. Over the last few years, several novel technologies have developed in the field of DBS therapy. Current-based programming, interleaved programming, fractionated current, and directional current steering are important examples. The following sections will discuss these recent developments which are important advances in the field of DBS programming.

### 3.1. Interleaved Programming

Interleaving strategy is applied when conventional programming techniques, such as bipolar, double monopolar, or tripolar settings, and use of alternative pulse widths and frequencies fail to achieve desired clinical results. Interleaving is also useful when stimulation induced side effects are elicited at lower voltages. Interleaving consists of a rapid and alternate activation of two electrode contacts with two distinct voltages and pulse widths but with an identical frequency, up to maximum of 125 Hz in the Medtronic Activa system (interleaving not available with St Jude and Boston Scientific). Thus a limitation in modulation of frequency potentially interferes with simultaneous control of tremors and other motor symptoms as tremors tend to respond to a higher frequency [25]. Interleaving is not the same as simultaneous double monopolar stimulation as the pulses at each of the two contacts could be potentially offset by 4 ms (125 Hz equals 8 ms interpulse interval). With interleaving, an area of overlap that receives stimulation from both the electrical fields at double the frequency is seen and this area is speculated to contribute to stimulation induced chronic side effects. Interleaving is also useful when two contacts require different voltages for control of two different symptoms. For example, interleaving allowed treatment of tremors and bradykinesia through stimulation of the subregions of subthalamic nucleus and the adjacent zona incerta [26]. In another case, interleaving was used to deliver pulses to the ventral intermedius nucleus of the thalamus as well as the subthalamic nucleus region in a patient who presented with coexisting diagnosis of essential tremor and Parkinson's disease [27]. The main drawback of interleaving to keep in mind is the possibility of an increased battery drain which is a concern if Parkinson's disease patient symptoms of dystonia require high stimulation voltages and pulse widths [25].

### 3.2. Directional Stimulation

With the advent of directional lead technology, it is now possible to steer different shapes of current at the stimulation contact instead of providing the conventional spherical shape of current. A major advantage of this technology is steering current to the desired structures and avoidance of unintended stimulation of the neighboring anatomical structures. This new technology facilitates achievement of greater efficacy and fewer side effects [28]. This is especially desirable when small and complex brain regions are targeted [29], such as the pedunculopontine nucleus [30], or other fiber bundle targets, such as the medial forebrain bundle. Direct STN Acute (Aleva Neurotherapeutics SA) that incorporates six directional contacts with three directional contacts on each of the two levels was investigated in a recent pilot study of Parkinson's disease patients who underwent subthalamic nucleus lead implantation. This lead also had two omnidirectional electrodes proximal to the directional contacts. The directional contacts were each 1 mm × 1 mm in dimension, with a longitudinal spacing of 0.5 mm. The investigators compared the effects of directional stimulation to omnidirectional stimulation in an intraoperative setting, focusing specifically on the volume of tissue activated. They found that the volume of tissue activated with directional stimulation (4.2 mm^3^) was substantially lower compared to the omnidirectional stimulation (10.5 mm^3^). As a consequence, the therapeutic window was significantly wider (43% wider) and the side effects were much lower with directional stimulation [28]. Another parallel study tested a novel 32 contact lead (formerly Sapiens Steering Brain Stimulation BV, Eindhoven, the Netherlands, now called Medtronic Eindhoven Design Center). These contacts could be activated independently in clusters, allowing for directional steering of the stimulation field and directional recording of local field potentials. In this study, thresholds for therapeutic benefit and side effects determined intraoperatively in 8 patients with Parkinson's disease were noted to be increased and the therapeutic window widened [31]. Recently Vercise directional lead (Boston Scientific, Valencia, CA), which has eight-contact leads and a pulse generator capable of multiple independent current source, was tested in seven Parkinson's disease patients. This novel lead with four electrode levels had two middle level electrodes split into three segments spanning approximately 120 degrees each and ring shaped electrodes in the highest and the lowest level. An extended monopolar review session was performed during the first week after the placement of leads. The current thresholds for control of rigidity and stimulation induced adverse effects were determined using either directional or ring-mode settings. Similar to the previous two studies, the investigators reported an expansion of the therapeutic window with this novel system [32]. The benefits of directional stimulation were best appreciated when the lead was suboptimally placed and the therapeutic window was narrow, for example, when the subthalamic nucleus lead was laterally placed close to the internal capsule. While these results are promising, larger studies are warranted for further confirmation.

### 3.3. Current-Based Programming and Fractionalization of Current

For several years, DBS therapy involved the use of voltage based programming. However the fluctuations in the impedance at the level of electrode-tissue interface were noted to contribute to an instability of voltages delivered to the target neural tissue [33]. As a result, stimulation parameters required frequent adjustments especially during the initial programming period after the DBS lead has been placed. These undesirable fluctuations also led to the understimulation or overstimulation of the intended target. These factors prompted the development of current-controlled DBS that regulated the current delivered to the targeted neural tissue regardless of the impedance. With a constant-current device, the need for programming adjustments was expected to reduce and the outcomes of DBS programming were expected to be more reliable. In a randomized multicenter controlled study, a constant-current device was examined in Parkinson's disease patients who underwent bilateral subthalamic nucleus implantation [7]. Subjects participating received either immediate stimulation or a delayed stimulation which was initiated three months after surgery (control group). The primary outcome of the study was the mean increase in the amount of medication ON time, and it was significantly increased in the immediate stimulation group (4.27 h versus 1.77 h, *p* = 0.003). The immediate stimulation group also performed better than the control group in the off-medication/on-stimulation assessment of Unified Parkinson's Disease Rating Scale motor score (40% improvement in the immediate stimulation group). The study was not primarily designed to determine the frequency of programming adjustments or compare constant-current with constant-voltage neurostimulation. In another crossover study of 8 Parkinson's disease cases, patients were randomized to constant-current and constant-voltage setting at about two years after subthalamic nucleus DBS surgery [33]. In both groups, the improvements in the motor scores, the reduction in levodopa dose, and the quality of life improvement were equivalent. The study concluded that constant-current stimulation programming was not necessarily superior to constant-voltage stimulation.

An accurate DBS targeting is critical for successful control of motor symptoms, and a slight error in lead location can sometime significantly impact clinical outcomes. In these circumstances, the delivery of small amounts of stimulation to multiple contacts is desirable. Until now, the DBS system consisted of a single source stimulation device. In the recent VANTAGE study, a multiple source delivery of fractionalized currents (Vercise DBS system, Boston Scientific) was examined in 40 Parkinson's disease patients who underwent bilateral subthalamic nucleus DBS surgery [23]. The Vercise DBS lead consisted of 8 contact rings one above the other on each side; each contact was 1.5 mm in length with 0.5 mm spacing between the contacts. A fractionated current with a well-defined shape of the electrical field allowed an enhanced and reliable motor response with minimized stimulation induced side effects. Once the healthcare programmer identified the contact that provided the best clinical benefits, the current was fractionalized between the best and the next best contact. Patients were then sent home with an ability to make adjustments at a preset stimulation range. In this open label study, Parkinson's disease patients were noted to improve by nearly 60% when comparing the baseline UPDRS motor scores (37.4 ± 8.9) with the six months postoperative scores (13.5 ± 6.8). There were also improvements in the quality of life, increase in the time spent in the medication on state, and reduction of the overall dose of dopaminergic medications. These outcomes were regarded better in comparison to other DBS trials and the incidence of adverse effects was in the acceptable range. Thus fractionalization of current is an important contribution to advanced DBS programming that will be soon applied in many more clinical studies.

### 3.4. Closed Loop DBS

There is an increasing enthusiasm for the use of closed loop DBS or adaptive DBS which represents a real-time change of DBS parameters in response to underlying physiological signals. The real-time change enables a more efficient control of clinical symptoms and at the same time there is a lesser use of battery [34]. However several questions have been raised over the best possible underlying physiological signal. In Parkinson's disease, these signals could be potentially recorded from the cortex, basal ganglia, and the skin surface over the affected body part (e.g., surface EMG) [35]. Local field potentials (LFPs) recorded from the basal ganglia are promising markers. LFPs indicate the oscillatory activity of a neuronal population surrounding the recording electrode and are usually clustered into specific frequency bands. The beta band frequency (11–30 Hz) is regarded as antikinetic, contributing to the bradykinesia and freezing of gait [36], whereas gamma band frequencies (>60 Hz) have a prokinetic role [37]. Beta band oscillations recorded from the subthalamic nucleus are found to be modulated by dopaminergic medication [38] and electrical stimulation [39]. They have been found to correlate with movement preparation and execution [40], akinesia [41], and the freezing of gait [42]. In a proof-of-principle study, LFP-based adaptive DBS was investigated in 8 patients with advanced PD who underwent subthalamic nucleus DBS [43]. The investigators applied an arbitrary threshold to the LFP power recorded from the subthalamic nucleus with DBS programmed to switch off if the beta power fell below threshold. Adaptive DBS was found to lead to a 30% greater motor improvement compared to continuous DBS therapy. Another source of physiological signals for adaptive programming is the cortex. Cortical signals recorded with electrocorticography have been frequently used for detection of seizures. In a primate model of Parkinson's disease, there was alleviation of akinesia when short stimulation trains (130 Hz) were delivered to the globus pallidus internus at fixed latency following an action potential recorded from the primary motor cortex area [44]. In another example of Parkinson's disease patient, there was improvement in rigidity when the phase amplitude coupling between beta and gamma oscillations of the cortical signals was observed to be decreased [45]. Closed loop stimulation will be increasingly utilized as the clinical advantages become established in patients with Parkinson's disease.

### 3.5. Applying Novel DBS Pulse for Programming

The conventional DBS therapy is a continuous delivery of charge-balanced, square waveform, cathodic pulse at specific voltages, and pulse widths that are within the limits of FDA recommended safety guidelines (30 mC/cm^2^). The square waveform DBS pulse has an active high-amplitude, short-duration stimulation phase, and an exponential passive low-amplitude, long-duration recharge phase that prevents tissue damage. However Hofmann et al. found that when the initial cathodic phase was followed by a short gap of time prior to introduction of an anodic phase, the neural activation and entrainment became more effective [46]. Foutz and McIntyre examined the effects of novel pulse shapes such as Gaussian, exponential, triangular, and sinusoidal pulses in both intracellular and extracellular environment to find that neural effects were elicited at lower energy consumption [47]. However, using biphasic pulse DBS therapy in which charge-balanced square-wave pulse with active recharge was used for patients with Parkinson's disease led to greater clinical benefits but at the cost of an increased battery drain [48]. Nevertheless, these applications of novel pulse shapes are promising and warrant further testing in a clinical population.

## 4. Guided Programming

### 4.1. Computer Guidance

Until now, DBS programming is mostly a time consuming and labor intensive manual process. DBS programming is also inconvenient to many patients as the DBS centers are few for meeting the needs of an increasing number of patients (more than 140,000 DBS surgeries performed worldwide) and often far away from a patient's home [5]. The complexities involved in clinical programming are perceived as burdensome by many healthcare providers [49]. Therefore, there are growing efforts to develop computer guided programming in conjunction with a sensor-based technology for feedback. Motion sensor-based feedback has been found to result in a better clinical outcome compared to subjective assessment [50]. The feasibility of computer guided DBS programming and automated motion sensor-based assessment, requiring minimal physician involvement, has been examined in a pilot study [5]. In this study, once the software performed the initial monopolar review, multiple iterations were conducted based on the automated feedback. The software then applied an algorithm to determine the final stimulation settings required to achieve control of symptoms and at the same time minimize the side effects and the battery usage [49]. The investigators concluded that significant improvement in tremors and bradykinesia could be achieved with minimal clinician involvement. Even though these findings are promising, they will require further confirmation in the clinical settings.

### 4.2. Visual Guidance

DBS programming is regarded as an “empirical” and “blind” technique. The clinician empirically inputs the electrical parameters and awaits the patient response as the output. Over the last few years, computational models have been developed that incorporate individual patient neuroanatomy to facilitate visual programming. Recently, these models were tested with an iPad application interface (ImageVis3D Mobile) that provided a mobile environment for a visual feedback on the interaction of the stimulation parameters with the surrounding anatomy [51]. Aside from clear advantage in visual feedback, programming time reduced from over 4 hours to less than 2 minutes (>99% saving in time) with computational model [51]. Diffusion tensor imaging and other advanced MRI sequences can potentially contribute to improved visually guided programming. Commercial programming platforms available through the Boston Scientific (Boston Scientific Guide DBS) and Medtronic (Medtronic Optivise) should be soon available for visually guided programming [24].

In summary, the success of DBS is dependent on numerous factors including appropriate selection of patients, appropriate patient expectations, accurate placement of DBS lead, and a thorough programming to identify the optimal stimulation parameters. Although there are general guidelines available for programming, there are no protocols that are validated and clearly established. Identifying the lead type, electrode configuration, impedance in the electrical system, and battery check are key elements for programming visits. There are growing efforts to advance the current approach to DBS programming. With the advent of fractionated current technology, it is now possible to distribute current to electrodes in fractions for a broader capture of motor symptoms. Directional lead steers different shapes of current to stimulate the desired structures and avoid unintended stimulation of the neighboring anatomical structures. Since DBS programming is a time consuming and labor intensive manual process, there is increasing interest to develop computer and visually guided protocol. Programming is also not a comfortable experience for the patient as it requires frequent clinic visits and programming facilities may not necessarily be close to the patient home. However remote and Internet based programming are likely to resolve these issues in the near future.

## Figures and Tables

**Table 1:** Clinical effects of stimulating individual targets employed in Parkinson's disease depend on spread of current.

	Optimal location stimulation	Medial spread of current	Lateral spread of current	Anterior spread of current	Posterior spread of current	Dorsal or superior spread of current	Ventral or inferior spread of current
*Subthalamic nucleus*							
Anatomical structure stimulated	Dorsolateral aspect of subthalamic nucleus (motor territory)	Cranial nerve III Red nucleus Limbic aspect	Corticospinal tract/internal capsule Frontal eye field fibers of internal capsule	Corticospinal tract/internal capsule Hypothalamus	Medial lemniscus	Internal capsule Thalamus Zona incerta	Substantia nigra reticulata Internal capsule fibers
Clinical effects	Control of tremors, bradykinesia, and rigidity	Diplopia, eye deviation, dizziness Sweating, nausea, paresthesia, warm sensation Personality change Depression, impulsivity	Facial pulling Limb contraction contralateral side Contralateral deviation of gaze	Facial pulling Limb contraction contralateral side Autonomic symptoms Sweating, nausea	Paresthesia (tingling, electrical sensation, numbness)	Contralateral muscle contraction Improvement of dyskinesia/tremor Improvement of dyskinesia/tremor	Mood changes Depression Muscle contractions
*Globus pallidum*							
Anatomical structure stimulated	Posteroventral aspect of globus pallidum	Internal capsule posterior limb	Globus pallidum externus Putamen	Globus pallidum externus Putamen	Internal capsule posterior limb	Globus pallidum externus	Optic tract
Clinical effects	Reduction of bradykinesia, rigidity, and dystonia	Contralateral muscle contractions	No effect	No effect	Contralateral muscle contractions	Putamen No effect	Phosphenes
*Thalamus*							
Anatomical structure stimulated	Ventral intermedius nucleus located in the middle of thalamus	Medial aspect of nucleus, centromedian nucleus/parafascicular nucleus	Internal capsule posterior limb	Ventral oralis anterior Ventral oralis posterior	Ventral caudalis nucleus	Dorsal aspect of thalamic nucleus Internal capsule fibers	Zona incerta Medial lemniscus Brachium conjunctivum
Clinical effects	Tremor control	Dysarthria	Dysarthria Contralateral muscle contractions	No effect Reduction of tremor	Paresthesia	No effect Dysarthria	Improvement of tremor/dyskinesia Paresthesia ataxia

**Table 2:** DBS system battery life and impedance limit.

DBS system company model	Battery type	Battery life (average)	Impedance limit for open circuit in ohms.	Impedance limit for short circuit in ohms
Medtronic Soletra™ (no longer manufactured)	Single chamber Nonrechargeable battery	3 to 5 years	If it is >2,000 ohms for bipolar and corresponding monopolar configurations and current is less than 10 uamps then it is likely open circuit.	<250 ohms

Medtronic Kinetra™ (no longer manufactured)	Dual chamber Nonrechargeable battery	3 to 5 years	If it is >4,000 ohms for bipolar and corresponding monopolar configurations and current is less than 10 uamps then it is likely open circuit.	<250 ohms

Medtronic Activa SC	Single chamber Nonrechargeable battery	3 to 5 years	If it is 5000–9000 ohms for bipolar and corresponding monopolar configurations suspect possible threatened open circuit. If it is >10000 ohms for bipolar and corresponding monopolar configurations then there is high suspicion of open circuit. If it is >40000 likely there is fracture in system.	<250 ohms

Medtronic Activa PC	Dual chamber Nonrechargeable battery	3 to 5 years	Same as Activa SC.	<250 ohms

Medtronic Activa RC	Dual chamber Rechargeable battery	8 years	Same as Activa SC.	<250 ohms

St Jude Libra™ (no longer manufactured)	Single chamber Nonrechargeable battery	3 to 5 years	High (31) is an open circuit.	

Abbott Infinity 5™	Dual chamber Nonrechargeable battery	3 to 4 years	>3000 Ohms.	

Abbott Infinity 7™	Dual chamber Nonrechargeable battery	4 to 5 years	>3000 Ohms.	
